# High-Contact Object and Surface Contamination in a Household of Persons with *Monkeypox Virus* Infection — Utah, June 2022

**DOI:** 10.15585/mmwr.mm7134e1

**Published:** 2022-08-26

**Authors:** Jack A. Pfeiffer, Abigail Collingwood, Linda E. Rider, Faisal S. Minhaj, Audrey M. Matheny, Chantal Kling, Andrea M. McCollum, Leisha D. Nolen, Clint N. Morgan

**Affiliations:** ^1^Epidemic Intelligence Service, CDC; ^2^Utah Department of Health and Human Services; ^3^CDC 2022 Multi-National Monkeypox Response.

In May 2022, the Salt Lake County Health Department reported two real-time polymerase chain reaction (PCR)–confirmed travel-associated cases of monkeypox to the Utah Department of Health and Human Services (UDHHS). The two persons with monkeypox (patients A and B) lived together without other housemates. Both persons experienced prodromal symptoms (e.g., fatigue and body aches). Eight days after symptom onset, patient A experienced penile lesions; lesions spread to the lips, hands, legs, chest, and scalp by day 10. Patient B experienced prodromal symptoms 8 days after illness onset of patient A; patient B experienced a lesion on the foot which spread to the leg and finger by day 11. Although both patients had lesions in multiple anatomic areas, the overall number of lesions was small, and lesions varied in presentation from “pimple-like” or ulcerated, to characteristically well-circumscribed and centrally umbilicated. Both patients had mild illness. The time from symptom onset to resolution was approximately 30 days for patient A and approximately 22 days for patient B.

To assess the presence and degree of surface contamination of household objects contacted by monkeypox patients, UDHHS swabbed objects in the home of the patients. The patients identified high-contact objects and surfaces for sampling; the patients also described cleaning and disinfection activities performed within the home during their illness and locations within the home where they spent substantial amounts of time while ill. The patients had isolated at home for 20 days before their home was entered for sampling. The patients were still symptomatic at the time UDHHS collected specimens from their home. The temperature in the two-story home ranged from 69°F (20.6°C) to 75°F (23.9°C) during their period of isolation. CDC monkeypox-specific cleaning and decontamination guidance (*1*) was shared with the occupants at the time the home surfaces were swabbed.

UDHHS personnel entered the residence discreetly wearing recommended personal protective equipment (*2*). They performed targeted environmental sampling using published methods (*3*). Specimens were obtained from 30 objects in nine areas of the home and were transported to the Utah Public Health Laboratory for shipment to CDC where they were processed and tested with both nonvariola Orthopoxvirus and West African *Monkeypox virus*–specific real-time PCR assays (*4*,*5*). Viral culture was only pursued if the qualitative PCR result was positive.* This activity was reviewed by CDC and was conducted consistent with applicable federal law and CDC policy.^†^

Among the 30 specimens, 21 (70%) yielded positive real-time PCR results, including those from all three porous items (i.e., cloth furniture and blankets), 17 of 25 (68%) nonporous surfaces (e.g., handles and switches), and one of two mixed surface types (i.e., chair) (Table). No specimen yielded a positive viral culture result. During the period of isolation both residents of the home reported showering once or twice each day, performing hand hygiene approximately 10 times daily, laundering bedding and clothing weekly, and performing routine household cleaning (e.g., mopping and daily use of a multisurface spray on most high-contact surfaces). The cleaning spray used was not listed on the Environmental Protection Agency’s List of Disinfectants for Emerging Viral Pathogens.^§^

**TABLE:** Results of testing for evidence of *Monkeypox virus* on high-contact objects and surfaces swabbed in a household of persons with monkeypox — Utah, June 2022

Surface type	Object/Surface	Room	Material type	Visibly soiled	Average* OPXV PCR Ct value	Average* West African clade MPXV PCR Ct value	Real-time PCR interpretation^†^	Culture result
Porous	Couch and blanket	Living room	Fabric	No	32.5	32.9	Positive	Negative
Porous	Chaise lounge	Bedroom	Cloth	No	35.2	35.3	Positive	Negative
Porous	Blankets (bed, top)	Bedroom	Fleece	No	34.3	36.1	Positive	Negative
Nonporous	Light switch	Bathroom 1	Plastic	No	37.1	35.6	Positive	Negative
Nonporous	Toilet handle	Bathroom 1	Metal	No	38.0	36.7	Positive	Negative
Nonporous	Toilet seat	Bathroom 1	Plastic	Yes	31.0	30.5	Positive	Negative
Nonporous	Refrigerator handle/ Ice dispenser	Kitchen	Stainless steel	No	35.9	36.9	Positive	Negative
Nonporous	Coffee maker	Kitchen	Stainless steel	No	36.5	36.4	Positive	Negative
Nonporous	Light switch	Bathroom 2	Plastic	No	36.4	37.3	Positive	Negative
Nonporous	Shower door handle	Bathroom 2	Plastic	No	35.3	36.3	Positive	Negative
Nonporous	Toilet handle	Bathroom 2	Metal	No	36.9	36.8	Positive	Negative
Nonporous	Sink handle	Bathroom 2	Metal	No	30.9	31.5	Positive	Negative
Nonporous	Faucet handle	Bathroom 3	Metal	No	28.7	29.6	Positive	Negative
Nonporous	Shower attachment	Bathroom 3	Unknown	No	36.2	37.1	Positive	Negative
Nonporous	Light switch	Landing	Plastic	No	36.9	37.7	Positive	Negative
Nonporous	Banister	Landing	Wood	No	33.5	33.2	Positive	Negative
Nonporous	Computer mouse	Office	Plastic	No	36.2	35.5	Positive	Negative
Nonporous	Keyboard	Office	Plastic	No	34.9	35.2	Positive	Negative
Nonporous	Medicine tube	Office	Plastic	No	33.7	34.5	Positive	Negative
Nonporous	Oven knobs	Kitchen	Stainless steel	Yes	ND	ND	Negative	NT
Nonporous	Door handle	Bathroom 2	Metal	No	ND	39.0	Inconclusive	NT
Nonporous	Blind pull	Office	Wood	No	37.8	ND	Inconclusive	NT
Nonporous	Computer mouse	Dining room	Plastic	No	36.3	37.1	Positive	Negative
Nonporous	Dining room chair	Dining room	Leather	No	37.5	38.5	Inconclusive	NT
Nonporous	Microwave handle	Kitchen	Stainless steel	No	37.5	37.6	Inconclusive	NT
Nonporous	Television remote	Living room	Plastic	No	37.3	37.3	Inconclusive	NT
Nonporous	Thermostat	Living room	Plastic	No	38.1	37.3	Inconclusive	NT
Nonporous	Remote	Bedroom	Plastic	No	38.2	ND	Inconclusive	NT
Mixed	Desk chair	Office	Imitation leather/Plastic	No	34.0	34.4	Positive	Negative
Mixed	Pillow/Desk chair	Dining room	Flannel/Wood	No	37.4	38.4	Inconclusive	NT

**Abbreviations:** Ct = cycle threshold; MPXV = *Monkeypox virus*; ND = not detected; NT = not tested; OPXV = *Orthopoxvirus*; PCR = polymerase chain reaction.

* PCR assays were run in duplicate for each specimen.

^†^ In both PCR assays, Ct values of 37–40 are considered inconclusive. Because of differential sensitivities between the real-time PCR assays, interpretation of discordant results are as follows: positive + inconclusive = positive; negative + inconclusive = inconclusive; positive + negative = inconclusive.

*Monkeypox virus* DNA was detected from many objects and surfaces sampled indicating that some level of contamination occurred in the household environment. However, the inability to detect viable virus suggests that virus viability might have decayed over time or through chemical or environmental inactivation. Although both patients were symptomatic and isolated in their home for >3 weeks, their cleaning and disinfection practices during this period might have limited the level of contamination within the household. These data are limited, and additional studies are needed to assess the presence and degree of surface contamination and investigate the potential for indirect transmission of *Monkeypox virus* in household environments. 

*Monkeypox virus* primarily spreads through close, personal, often skin-to-skin contact with the rash, scabs, lesions, body fluids, or respiratory secretions of a person with monkeypox; transmission via contaminated objects or surfaces (i.e., fomites) is also possible.^¶^ Persons living in or visiting the home of someone with monkeypox should follow appropriate precautions against indirect exposure and transmission by wearing a well-fitting mask, avoiding touching possibly contaminated surfaces, maintaining appropriate hand hygiene, avoiding sharing eating utensils, clothing, bedding, or towels, and following home disinfection recommendations.**^,^^††^

## Footnotes

* https://www.cdc.gov/poxvirus/monkeypox/clinicians/prep-collection-specimens.html (Accessed August 3, 2022).

† 45 C.F.R. part 46.102(l)(2), 21 C.F.R. part 56; 42 U.S.C. Sect. 241(d); 5 U.S.C. Sect. 552a; 44 U.S.C. Sect. 3501 et seq.

§ https://www.epa.gov/pesticide-registration/disinfectants-emerging-viral-pathogens-evps-list-q#evps (Accessed August 10, 2022).

¶ https://www.cdc.gov/poxvirus/monkeypox/transmission.html (Accessed August 15, 2022).

** https://www.cdc.gov/poxvirus/monkeypox/clinicians/infection-control-home.html (Accessed August 8, 2022).

†† https://www.cdc.gov/poxvirus/monkeypox/specific-settings/home-disinfection.html (Accessed August 15, 2022).

## References

[1] CDC. Disinfecting home and other non-healthcare settings. Atlanta, GA: US Department of Health and Human Services, CDC; 2022. Accessed August 8, 2022. https://www.cdc.gov/poxvirus/monkeypox/specific-settings/home-disinfection.html

[2] CDC. Infection prevention and control of monkeypox in healthcare settings. Atlanta, GA: US Department of Health and Human Services, CDC; 2022. Accessed August 3, 2022. https://www.cdc.gov/poxvirus/monkeypox/clinicians/infection-control-healthcare.html

[3] Morgan CN, Whitehill F, Doty JB, Environmental persistence of monkeypox virus on surfaces in household of person who had travel-associated infection, Dallas, Texas, USA, 2021. Emerg Infect Dis 2022;28. 10.3201/eid2810.221047 35951009 PMC9514334

[4] Li Y, Olson VA, Laue T, Laker MT, Damon IK. Detection of monkeypox virus with real-time PCR assays. J Clin Virol 2006;36:194–203. 10.1016/j.jcv.2006.03.012 16731033 PMC9628957

[5] Li Y, Zhao H, Wilkins K, Hughes C, Damon IK. Real-time PCR assays for the specific detection of monkeypox virus West African and Congo Basin strain DNA. J Virol Methods 2010;169:223–7. 10.1016/j.jviromet.2010.07.012 20643162 PMC9628942

